# Optical finger phantom with realistic optical properties

**DOI:** 10.1364/BOE.576585

**Published:** 2025-11-13

**Authors:** Markus Wagner, Christian Blum, Alwin Kienle, Florian Foschum

**Affiliations:** 1 Institut fuer Lasertechnologien in der Medizin und Meßtechnik an der Universität Ulm, Helmholtzstraße 12, 89081 Ulm, Germany; 2 University Ulm, Helmholtzstraße 12, 89081 Ulm, Germany

## Abstract

Existing optical phantoms often do not represent realistic optical and geometrical properties. This study aimed to fabricate a homogeneous silicone finger phantom that closely mimics the reflectance and transmittance characteristics of a human finger by precisely adjusting the absorption and reduced scattering coefficients in the visible wavelength range. The absorption and reduced scattering coefficients of a human finger were determined using a custom inverse model tailored for an integrating sphere system designed for cylindrical media illuminated along the barrel. To reproduce the retrieved optical properties in silicone, a reference database was created by characterizing the absorption spectra of 15 pigments dispersed in a silicone matrix. An automated fitting algorithm identified five suitable absorbing pigments, and their required concentrations were calculated to match the target absorption spectrum. The reduced scattering coefficient was independently controlled by varying the concentration of zirconium dioxide particles. An alginate mould was used to capture the finger geometry, ensuring anatomical accuracy of the phantom. The fabricated silicone finger phantom closely matched the human finger in both transmittance and reflectance, as well as in its anatomical shape. The Δ*E*_2000_ value between the reflectance spectra of the human and silicone fingers was found to be 0.85. Under transmittance-mode illumination, light propagation within the silicone phantom agreed well with that of a human finger, both in visual appearance and in spatial light distribution. A method was developed to fabricate silicone finger phantoms with accurately matched optical and anatomical properties.

## 1. Introduction

In biomedical research, tissue phantoms are used to replicate the physical and optical properties of biological tissues. Their main applications include system testing, optimization of signal quality and to evaluate and compare the performance of different systems. [1]. A comprehensive review of phantom types in biomedical imaging and fabrication criteria is provided by Pogue and Patterson [1] and Hacker et al. [2]. In biomedical optics, materials such as Agar, polyvinyl alcohol, copolymer in oil, silicone and epoxy resin are used for tissue mimicking phantoms [3–8]. Recently, silicone has been increasingly used in the relevant literature [9–11]. The primary advantage of silicone lies in its long-term stability and relative ease of production. By adding scattering particles or an absorbing medium to the silicone, it is possible to adjust its optical properties, specifically the absorption coefficient, here denoted as $\mu_{\text{a}}$ and the reduced scattering coefficient, here denoted as $\mu_{\text{s}}^{\prime }$ . Its elastic nature also makes silicone particularly suitable for anatomically shaped phantoms, such as fingers [12–15]. However, due to its higher acoustic attenuation and limited tunability of acoustic properties, silicone is less suitable for photoacoustic applications [2].

To effectively mimic human tissue, it is essential to understand the underlying optical behavior of skin. Human skin is known to scatter and absorb light due to the presence of various components within the tissue, including collagen and elastin fibers. The degree of scattering depends on the composition and size of the constituent microstructure within the tissue. The absorption of light within the visible spectrum is predominantly influenced by blood and melanin. However, other factors, such as bilirubin and collagen, also play a role in this process [16,17]. Several approaches to build skin mimic phantoms have already been documented in the literature to simulate tissue properties [9–11]. They used a black absorber to mimic the absorption value at one wavelength and did not take the wavelength dependent absorption of the skin into account. Moffitt et al. and Pekar and Patterson mimicked a broader wavelength range in Polyurethane or Epoxy resin [18,19]. Dyes have been used, but concerns have been raised about their stability. Jansen et al. produced epoxy resin phantoms and added up to 20 pigments to mimic different absorption and scattering properties of biological tissue, achieving a very good alignment spectrum from 370 to 950 nm and showing a good alignment between aimed and real spectra [20]. A phantom with a reflectance similar to skin was built. There the real geometrical shape and the ambiguity of a reflectance spectrum in $\mu_{\text{a}}$ and $\mu_{\text{s}}^{\prime }$ was not taken into account. To create even more realistic phantoms, it is important to use accurate $\mu_{\text{a}}$ and $\mu_{\text{s}}^{\prime }$ . Combined with precise geometric dimensions, this enables a more accurate representation of measurement scenarios. For instance, a finger phantom with realistic optical and geometric properties could be used for vital parameter detection in the finger. Such realistic phantoms generally provide controlled, reproducible test conditions for imaging system development, sensor calibration, prosthetic research and regulatory compliance. They bridge the gap between theoretical models and clinical applications by providing a known reference for comprehensive optical measurement validation.

The objective of the present research is to create a homogeneous optical silicone finger phantom that mimics the transmittance and reflectance of a finger with realistic absorption and scattering coefficients in the visible wavelength range. We determined $\mu_{\text{a}}$ and $\mu_{\text{s}}^{\prime }$ of a human finger using a novel inverse model specifically designed for integrating sphere measurements on a human finger, allowing for separation of $\mu_{\text{a}}$ and $\mu_{\text{s}}^{\prime }$ from reflectance and transmittance data. The reduced scattering coefficient of the phantom was adjusted using zirconium oxide pigments, while absorption was adjusted through five pigments. Anatomical fidelity was achieved by casting the phantom in a mold derived from an actual human finger. This work represents an initial step towards developing more advanced optical phantoms, with the current focus on validating the concept in the visible spectral range.

## 2. Material and methods

### 2.1. Phantom material

**Fig. 1.**
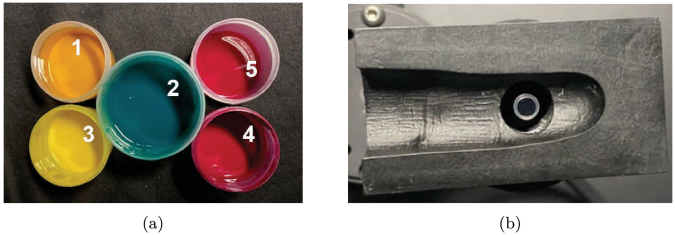
(a): The five pigments used in the prepared pigment preconcentrations. Their corresponding absorption spectra are presented in Fig. 3. (b): 3D-printed finger holder designed to ensure reproducible finger positioning for consistent imaging. Transmittance images were acquired using a 5 mm liquid light guide illuminated by a halogen lamp.

The silicone used as the base material was Elastosil M 4641 A/B (Wacker Chemie AG, Germany). Zirconium oxide (ZrO_2_) nanoparticles (US Research Nanomaterials, Inc.) with a size distribution of *d*_10_ = 0.131 *μ*m, *d*_50_ = 0.871 *μ*m and *d*_90_ = 3.228 *μ*m were employed to adjust the scattering properties of the phantom [21]. To tailor the absorption characteristics, a dataset comprising the absorption spectra of 15 organic and inorganic pigments was created. The organic pigments selected possessed a blue wool scale rating of at least 7, while the inorganic pigments reached a rating of 8, ensuring high lightfastness across all materials [22]. For each pigment, preconcentrations in silicone were prepared. Figure 1(a) shows the preconcentrations of the five pigments used in the finger phantom. These pigments are: (1) Eisenoxid Gelb-Orange (Kremer Pigmente GmbH, Germany), (2) Heliogen Grün Dunkel (Kremer Pigmente GmbH, Germany), (3) Cromophtal Yellow K 0990/K (BASF SE, Germany), (4) CinquasiaPink K 4410 (BASF SE, Germany) and (5) Cinquasia Magenta K 4535/K (BASF SE, Germany). Subsequently, the absorption spectra of the preconcentrations were determined by preparing test samples with different pigment concentrations in a scattering matrix containing zirconium oxide particles and measuring their optical properties using an integrating sphere setup. The absorption coefficient of the preconcentration was calculated by subtracting the control sample absorption from pigmented samples and dividing by the pigment concentration [23]. Intrinsic pigment scattering was neglected due to low concentrations used. A fitting procedure was applied to determine the precise pigment concentrations needed for the phantom. This fitting was performed using the lsqcurvefit function in MATLAB 2023a. Pigment concentrations were iteratively optimized to minimize differences between target and fitted absorption spectra. To ensure robustness and avoid local minima, the fitting process was repeated 30 times with different initial conditions. The set of concentrations with the lowest chi-squared error was selected for phantom fabrication.

### 2.2. Manufacturing process and moulding

The manufacturing process was based on a multi-step mixing procedure combined with a double asymmetric vacuum centrifuge (SpeedMixer DAC 800, Hauschild Engineering, Hamm, Germany), as described by Wagner et al.[23]. Two distinct phantom geometries were produced using the pigmented silicone: slabs and a realistic finger. The slabs were fabricated with varying thicknesses (1, 2, 4 and 6 mm) and a diameter of 35 mm. For slab fabrication, two acrylic glass plates and a 3D-printed mould were employed, following the method previously reported by Wagner et al.[23]. These slabs were subsequently used to determine the optical properties of the phantom material via an integrating sphere measurement setup. The second phantom, representing a human finger, was fabricated to match the dimensions of a real finger. The mould was created using alginate (TFC Alginat PREMIUM, Trollfactory, Germany) as the base material, with a real finger serving as the template. Alginate was mixed with water at a ratio of 1:3 and allowed to cure with the finger immersed. After approximately 10 minutes of curing, the finger was carefully removed and the resulting mould was filled with the pigmented silicone.

### 2.3. Integrating sphere measuring setup and inverse model

The reflectance, transmittance and optical properties in this study were characterized using an integrating sphere-based setup developed by Foschum et al. [24] and Bergmann et al. [25]. The setup is a lab version of the SphereSpectro 150H (Gigahertz Optik GmbH, Germany). The 3D-printed integrating sphere is barium sulfate coated, has an inner diameter of 150 mm and the measurable wavelength range extends from 400 to 1550 nm. The evaluation method was designed to determine the absorption coefficient $\mu_{\text{a}}$ and the reduced scattering coefficient $\mu_{\text{s}}^{\prime }$ of a slab and is based on Monte Carlo simulations and an analytical model [24]. The asymmetry factor *g* was set to 0.75 assuming a Henyey-Greenstein scattering phase function for all evaluations as only optically thick samples were used. The refractive index of the applied silicone was measured using an ellipsometer (SENresearch 4.0, SENTECH) and is shown by Wagner et al. [23]. These values are wavelength-dependent, ranging from 1.43 to 1.4 in the visible wavelength range. All measurements presented in this work were repeated three times to ensure reproducibility.

The optical properties of a human finger were assessed also using the integrating sphere measuring setup. However, a new look-up table (LUT) was generated by modeling the finger as a homogeneous cylinder illuminated perpendicular to its axis in the Monte Carlo simulation. This simulation, based on the radiative transport equation, accurately represents light propagation within the cylindrical geometry. Photons exiting the cylinder were subsequently evaluated to determine whether they entered the integrating sphere. The diameter of this cylinder corresponded to the thickness of the finger at the designated measuring location. The thickness of the used finger at the measuring site was found to be 13.8 mm and was measured with an micrometer screw. This LUT was used to determine the optical properties of the finger from its reflectance and transmittance. At short wavelengths, where the transmittance approaches very low values, the measurement of reflectance gives rise to an ambiguity in the values of $\mu_{\text{a}}$ and $\mu_{\text{s}}^{\prime }$ . For this case, the evaluation process was modified to calculate only the absorption coefficient $\mu_{\text{a}}$ while using a predefined reduced scattering coefficient $\mu_{\text{s}}^{\prime }$ . The wavelength-dependent $\mu_{\text{s}}^{\prime }$ was defined based on the zirconium oxide scatterer characterized in Sec. 2.1. The scatterer concentration was adjusted so that its $\mu_{\text{s}}^{\prime }$ matched the predetermined value of the finger at 700 nm. Subsequently, $\mu_{\text{a}}$ was calculated using the reflectance of the finger together with the predefined $\mu_{\text{s}}^{\prime }$ , employing the lookup table (LUT).

### 2.4. Photo box

To capture realistic images of both the phantom and human fingers, a custom-built photo box was used, as described by Hevisov et al. [26]. The interior of the box was lined with low-reflective black stage fabric to suppress stray light and define the imaging area. A rotatable multi-axis system, constructed from aluminum extrusion profiles, allowed flexible positioning. A Nikon D7500 DSLR camera equipped with a Nikon AF-S Micro Nikkor lens 60 mm was mounted perpendicular to the finger, while a custom-built LED white light source was positioned at a 45^∘^ angle. To further minimize stray illumination, an aperture was placed in front of the light source to confine the light field to the region of interest. To ensure reproducible finger positioning, a 3D-printed finger holder with anatomically realistic geometry was developed. The finger shape was initially captured using a 3D scanning sensor (ZEISS COMET). Based on the resulting 3D model, an inverse mould incorporating a fiber holder for transmittive illumination was designed and printed using a stereolithography (SLA) printer (Sonic Mighty Revo 14K, Phrozen), as shown in Fig., 1(b). The mould was printed using BASF Ultracur3D RG 35 black, a biocompatible photopolymer resin. For transmittance illumination, a liquid light guide with a 5 mm diameter was positioned identically to the setup used in the integrating sphere-based transmittance measurements. Illumination was provided by a custom-built halogen lamp. A schematic representation of the experimental setup is presented in Fig. S1.

## 3. Results

### 3.1. Optical properties of a finger

The aim of the present study was to create a phantom with spectrally resolved reflectance and transmittance equivalent to those of a real human finger in the visible spectrum, in order to replicate realistic light propagation characteristics. To achieve this, reflectance and transmittance measurements were performed on the little finger of a healthy 30-year-old male with Fitzpatrick skin type I, using an integrating sphere measurement system [27]. Subsequent to this, the associated optical properties $\mu_{\text{a}}$ and $\mu_{\text{s}}^{\prime }$ of the human finger were determined. Measurements taken at multiple locations along the finger revealed significant variations in transmittance, particularly in regions of increased thickness or anatomical complexity (e.g., over joints or the fingernail). Due to the observed homogeneity, the palmar side between the fingernail and the first interphalangeal joint, characterized by moderate, uniform thickness (approx. 13.8 mm), was selected as the measurement site. It is important to note that the resulting phantom is specific to this anatomical region and is not intended to represent other finger segments or tissue types, which may differ substantially in both structure and optical behavior. Although the phantom targets the visible range (400–730 nm), optical properties of a human finger were determined from 400 to 1000 nm to demonstrate their plausibility across a broader wavelength range.

**Fig. 2.**
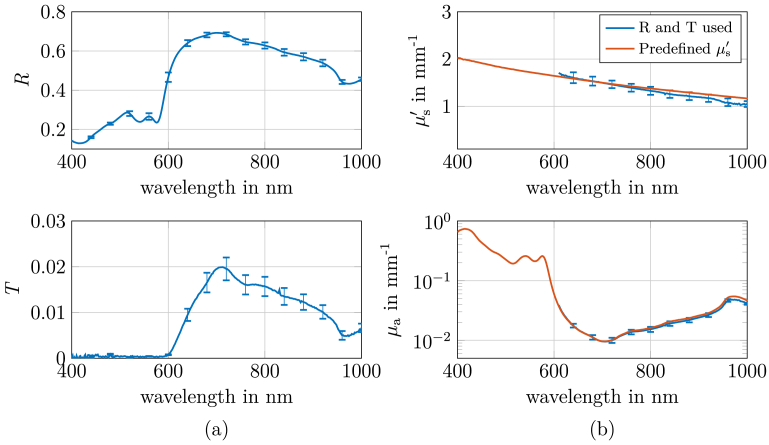
(a): Reflectance and transmittance spectra of a human little finger (measured between the fingernail and the first finger joint), acquired using an integrating sphere setup. (b): In blue: resulting $\mu_{\text{a}}$ and $\mu_{\text{s}}^{\prime }$ obtained from reflectance and transmittance using a cylindrical geometry model (600–1000 nm). In orange: absorption coefficient $\mu_{\text{a}}$ calculated from reflectance and a predefined $\mu_{\text{s}}^{\prime }$ based on ZrO_2_ particles. The predefined $\mu_{\text{s}}^{\prime }$ was set to match the value of the human finger at 700 nm.

The corresponding reflectance and transmittance spectra are presented in Fig. 2(a). Transmittance measurements showed negligible signal below 600 nm, limiting the direct determination of optical properties ( $\mu_{\text{a}}$ and $\mu_{\text{s}}^{\prime }$ ) to the spectral range between 600 and 1000 nm. Within this range, optical properties were derived using the lookup table (LUT) for a homogeneous cylindrical geometry. The resulting absorption and reduced scattering coefficients (Fig. 2(b)) show reasonable agreement with the general range of literature values for human skin [16,28], though direct quantitative comparison is limited by the variability in reported skin optical properties across different studies, anatomical locations and measurement techniques. To determine the optical properties in the spectral range from 400 to 1000 nm, a predefined reduced scattering coefficient $\left(\mu_{\text{s}}^{\prime }\right)$ was introduced to resolve ambiguities caused by the lack of transmittance data below 600 nm. Zirconium oxide particles were selected as scatterers due to their wavelength-dependent scattering properties, which closely resemble those of human finger tissue up to 600 nm. Their concentration was adjusted to match $\mu_{\text{s}}^{\prime }$ of the human finger at 700 nm. However, literature shows that tissue scattering increases more steeply with decreasing wavelength (600–400 nm) than observed for the used zirconium oxide particles. Consequently, the predefined $\mu_{\text{s}}^{\prime }$ may underestimate the actual tissue $\mu_{\text{s}}^{\prime }$ in the blue spectral range, theoretically leading to corresponding underestimation of $\mu_{\text{a}}$ during inverse modeling. Based on the predefined $\mu_{\text{s}}^{\prime }$ and the measured reflectance, $\mu_{\text{a}}$ was calculated across the full spectral range from 400 to 1000 nm, as shown by the orange line in Fig. 2. The close agreement in $\mu_{\text{s}}^{\prime }$ at 700 nm ensures a reliable estimation of $\mu_{\text{a}}$ in the red and near-infrared range, while possible deviations at shorter wavelengths (400–600 nm) should be considered when interpreting the results.

### 3.2. Pigment absorption adjustment

**Fig. 3.**
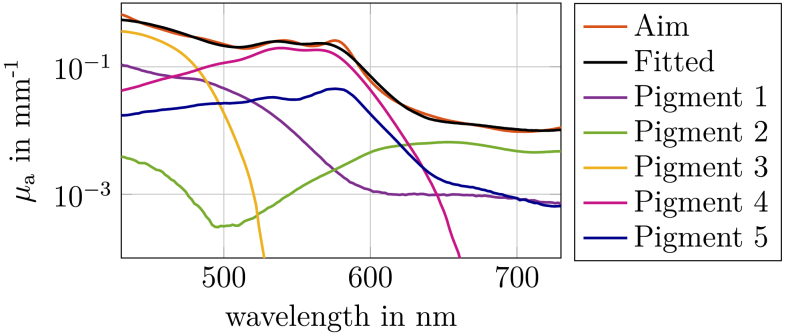
The figure compares the target absorption spectrum of the human finger (orange line in Fig. 2) with the best fitted absorption spectrum obtained using a combination of pigments. The fitted absorption curve includes contributions from five selected pigments, as well as the baseline absorption of silicone and zirconium oxide particles. The individual pigment curves represent their concentration-weighted absorption spectra. For clarity, the absorption contributions of silicone and ZrO_2_ are omitted from the plot.

In order to create a realistic silicone finger phantom in the visible spectrum, the absorption from Fig. 2(b) (orange curve) was selected as the target. Initially, all pigments from the absorption database (see Sec. 2.1) were fitted to this target spectrum, incorporating the baseline absorption of silicone and ZrO_2_. The optical properties of the silicone base material and the zirconium oxide particles are provided in Supplement 1 Fig. S2. The fitting procedure is described in detail in Sec. 2.1. Only five of the 15 available pigments were found to significantly contribute to the resulting spectrum. Subsequently, the absorption spectra of only these five pigments were refitted to the target and their concentrations were optimized accordingly. Figure 3 shows the resulting spectra of the weighted pigment concentrations. The contributions of the silicone matrix and ZrO_2_ particles are not shown but considered in the fitted absorption. A mean relative error of less than 9 % was achieved between the target and the fitted absorption spectra. The simulated reflectance of the resulting finger phantom, based on the fitted $\mu_{\text{a}}$ and predefined $\mu_{\text{s}}^{\prime }$ (see Sec. 2.3), was compared to the reflectance of the human finger. The resulting $\Delta E_{2000}$ between the simulated phantom and the human finger reflectance spectra was 0.21. For this calculation, both reflectance spectra were converted to the CIE L*a*b* color space using the CIE 2012 10^∘^ standard observer and a D65 illumination source [29]. ΔE is a well-established measure of color difference and provides a clear understanding of how closely we replicate the reflectance spectrum.

### 3.3. Optical properties of the phantom

**Fig. 4.**
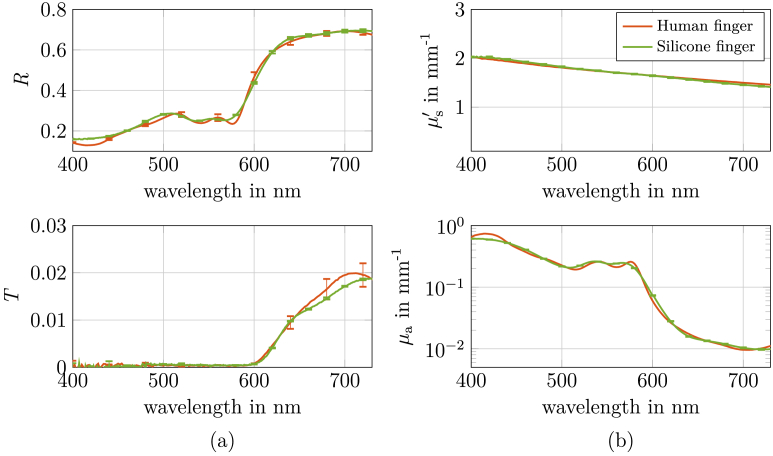
(a): Reflectance and transmittance of the fabricated silicone phantom finger, measured using an integrating sphere setup, in comparison to the target reflectance and transmittance of a human finger (see Fig. 2). (b): Absorption coefficient $\mu_{\text{a}}$ and reduced scattering coefficient $\mu_{\text{s}}^{\prime }$ of the silicone phantom, obtained from slab measurements, compared to the desired optical properties. All measurements were performed in triplicate. A color difference of $\Delta E_{2000}=0.85$ was achieved between the measured and target reflectance curves and the mean deviation in $\mu_{\text{a}}$ between the phantom and the real finger was 8.34 %.

For the fabrication of the phantom, the pigments were weighed according to the concentrations calculated in Section 3.2, thoroughly mixed and then cast into the respective moulds as described in Section 2.2. The silicone was subsequently cured at room temperature. The optical properties, in particular the transmittance (*T*) and reflectance (*R*), of the resulting silicone finger were measured using the integrating sphere setup and compared to those of a real human finger (see Fig. 4(a)). The results show a close agreement between the silicone phantom and the target object, indicating that our cylindrical model provides a good approximation of the optical properties of the finger. The color difference, quantified by $\Delta E_{2000}$ between the reflectance spectra of the real and the silicone finger, is 0.85. This value was determined by converting both reflectance spectra into the L*a*b* color space using the CIE 2012 10^∘^ observer function and a CIE D65 light source [29].

The optical properties of the phantoms, as depicted in Fig. 4(b), were determined by measuring slabs of varying thicknesses (1, 2, 4 and 6 mm). The absorption coefficient $\mu_{a}$ and the reduced scattering coefficient $\mu_{s}^{\prime }$ were subsequently stitched across the different thicknesses to achieve optimal reflectance (*R*) and transmittance (*T*) values. In comparison to the target optical properties of a human finger, the phantom exhibits a close match. The mean deviation in $\mu_{a}$ with respect to the absorption coefficient of the real finger is approximately 8.34 %. Furthermore, a high level of agreement was achieved between the predefined and the measured values of $\mu_{s}^{\prime }$ . The observed agreement in optical parameters suggests that the phantom fabrication process is characterized by reproducibility and process stability.

### 3.4. Photo comparison of the finger phantom and the target human finger

**Fig. 5.**
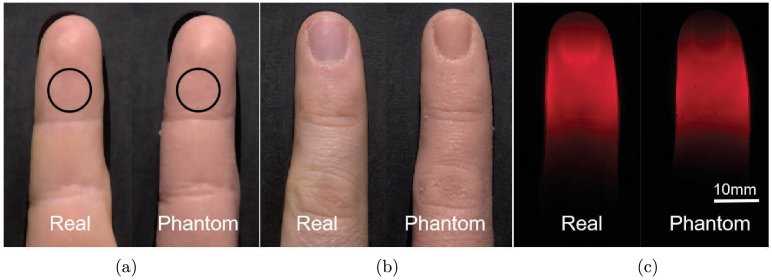
Photo comparison of a real finger (left) and a silicone finger (right). For panels (a) and (b), the samples were illuminated by an LED light panel positioned in front of the phantom. For panel (c), illumination was provided from beneath using a 5 mm diameter fiber connected to a halogen lamp, see Fig. 1(b). (a) Palmar (inner) side of the finger. The circle indicates the reflectance measurement area corresponding to the integrating sphere setup. (b) and (c) Dorsal (outer) side of the finger.

In addition to the measured reflectance *R*, transmittance *T* and the derived absorption coefficient $\mu_{\text{a}}$ and reduced scattering coefficient $\mu_{\text{s}}^{\prime }$ , the visual appearance of the fabricated silicone finger phantom was qualitatively evaluated. Photographs of both the palmar (inner) and dorsal (upper) sides of the finger were acquired, as illustrated in Fig. 5. In each panel, the real finger is positioned on the left and the silicone phantom on the right. Images were captured within a custom-built light box to ensure uniform illumination and the finger was secured in a 3D-printed holder to maintain consistent positioning across measurements, as described in Sec. 2.4. Figure 5(a) shows the palmar side of the finger. For privacy considerations, the image has been slightly blurred to obscure identifiable skin features. Within the marked circular region, which corresponds to the area where the reflectance measurements were performed, the color of the phantom closely resembles that of a real finger. Minor variations observed in the photographs are attributed to the inherent heterogeneity of skin texture and the complex multilayered structure of the finger, which influence visual appearance. In contrast, Fig. 5(b) compares the dorsal side, where a more pronounced color difference between the phantom and the real finger is evident. This discrepancy arises because the phantom’s optical properties were specifically tuned to match the reflectance characteristics of the palmar side, which differs structurally and chromatically from the dorsal side. Figure 5(c) presents images obtained under transillumination conditions, where the light source was repositioned beneath the finger, see Fig. 1(b). Despite the real finger’s complex multilayer anatomy—including features such as the fingernail, which are absent in the single-layer silicone phantom—the color distribution and light propagation patterns exhibit strong qualitative agreement. Notably, both the real and phantom fingers display a characteristic light halo and enhanced coloration within skin folds, demonstrating that the phantom faithfully reproduces key aspects of the optical response despite its simplified structure.

## 4. Discussion and conclusion

This proof-of-concept study demonstrates the fabrication of a silicone-based finger phantom that closely matches the spatially averaged optical properties of human tissue in the visible wavelength range. By carefully determining the absorption coefficient $\mu_{\text{a}}$ and reduced scattering coefficient $\mu_{\text{s}}^{\prime }$ of a human finger and closely matching them using absorbing and scattering pigments, we successfully replicated both the reflectance and transmittance spectra of a real human finger, as measured with an integrating sphere setup. This approach ensures that the phantom not only visually resembles human tissue but also accurately mimics its light transport characteristics, which is essential for biomedical optics applications.

The phantom successfully replicates real finger optical properties, as demonstrated by the close alignment in reflectance and transmittance measurements (see Fig. 4). This agreement of *R* and *T*, in addition to the optically photo similarity demonstrated in Fig. 5, serves to confirm the validity of the optical properties that have been determined. Moreover, the cylindrical geometry used to model the finger geometry in the Monte Carlo simulation proved adequate. The aim optical properties in this study, shown in Fig. 2, are based on measurements of the palmar side of a human finger with Fitzpatrick skin type I. The palmar side was chosen due to its relative homogeneity. Alternatively, the phantom’s optical properties could be adapted using measurements from the dorsal side or by determining properties for other fingers and Fitzpatrick skin types to extend its applicability. To ensure broader relevance, the general suitability of the cylindrical model should also be validated, as finger geometry can vary between individuals and measurement sites. Alternatively, instead of assuming a simplified cylindrical shape, the exact 3D geometry of the finger can be incorporated into the Monte Carlo simulation to more accurately determine the optical properties while accounting for subject-specific anatomical features.

A key strength of our approach is the implementation of a multipigment strategy, which enables precise spectral fitting across the visible range with low deviations of aim and fitted $\mu_{\text{a}}$ (see Fig. 3). This aligns with recent advances in phantom development, where the combination of multiple pigments allows for accurate emulation of tissue absorption and scattering properties over a broad spectrum [20]. Currently, our method is optimized for the visible spectral range (400–730 nm), with pigment selection focused on minimizing $\mu_{a}$ and $\mu_{s}^{\prime }$ deviations to get a close alignment of *R* and *T*. Extending the target wavelengths to a broader range increases the deviation between the desired and the phantom’s optical properties, as optimizing pigment composition becomes more challenging across a wider spectral range. However, for a finger phantom designed for a specific biomedical application, the wavelength range must be specifically tailored to the intended use case. For pulse oximetry, for example, wavelengths in the near-infrared region, particularly between 660 and 940 nm, are most relevant [30]. Therefore, it is necessary to adapt the optical properties of the phantom to match those of a human finger in this range, as illustrated in Fig. 2. While the reduced scattering coefficient of the phantom is already defined by the zirconium oxide particles in the NIR, adjusting the absorption coefficient requires the incorporation of additional pigments that replicate the absorption spectrum between 660 and 940 nm. Addressing this aspect will be the focus of future research.

The current phantom, made from silicone with a Shore A hardness of 28, is mechanically stiffer than human skin, which exhibits a Shore 00 hardness of 25 [15]. Adjusting the curing agent ratio may allow better alignment with the mechanical compliance of real tissue [15], improving tactile realism. Such enhancements combined with integration of vascular structures could enable to simulate PPG signals.

Anatomical fidelity was ensured through the use of an alginate mould, which captured the geometry of a human finger with high accuracy, see Fig. 5. This enables the phantom to replicate not only the optical but also the geometrical shape, supporting realistic probe positioning and contact in experimental setups. However, the phantom exhibits a slightly glossier surface compared to real skin, likely due to differences in microstructural roughness. Such surface characteristics can influence both the perceived color and the angular distribution of reflected light and may be particularly relevant for optical measurement techniques sensitive to surface reflectance components.

While the phantom’s homogeneous structure offers optical fidelity, it does not capture the natural layering and heterogeneity of human skin. Previous studies have shown that multilayered phantoms can more accurately represent the complex optical behavior of biological tissues, particularly for applications sensitive to superficial chromophores [8]. Future finger phantom production could address these limitations through advanced fabrication approaches based on MRI and ultrasound measurements, enabling phantoms with anatomically accurate structures incorporating different optical properties for each tissue layer. 3D-printed molds instead of alginate molds could enable precise control over layer thickness and geometry.

While the pigments exhibited high lightfastness, their long-term behaviour in combination with the silicone, particularly under varying conditions, remains to be validated. Further investigation is required into the long-term stability of both the silicone matrix and the embedded pigments.

Despite potential areas for improvement, our approach has successfully demonstrated the process for producing finger phantoms with realistic optical properties. The combination of optimized optical properties with 3D-molded anatomical geometry shows that silicone-based phantoms can accurately replicate both reflectance and transmittance characteristics. This methodology establishes a robust and adaptable foundation for developing next-generation optical phantoms.

## Supplemental information

Supplement 1 New Supplement Document with all Suplement Figures https://doi.org/10.6084/m9.figshare.30327376

## Data Availability

Data underlying the results presented in this paper are not publicly available at this time but may be obtained from the authors upon reasonable request.

## Supplemental document

See Supplement 1 for supporting content.
