# Supplementary material for: Optical finger phantom with realistic optical properties
Source: Biomed Opt Express. 2025 Nov 13;16(12):5150–60. doi: 10.1364/BOE.576585 (PMC12698079; doi:10.1364/BOE.576585)
Supplement: Supplementary file 1 [file boe-16-12-5150-s001.pdf]

Supplemental Document

Biomedical Optics EXPRESS

Optical finger phantom with realistic optical properties: supplement

MARKUS WAGNER,[1,2,*] CHRISTIAN BLUM,[1,2] ALWIN KIENLE,[1,2] AND FLORIAN FOSCHUM[1]

[1] *Institut fuer Lasertechnologien in der Medizin und Meßtechnik an der Universität Ulm, Helmholtzstraße 12, 89081 Ulm, Germany*
[2] *University Ulm, Helmholtzstraße 12, 89081 Ulm, Germany*
[*] *markus.wagner@ilm-ulm.de*

This supplement published with Optica Publishing Group on 13 November 2025 by The Authors under the terms of the Creative Commons Attribution 4.0 License in the format provided by the authors and unedited. Further distribution of this work must maintain attribution to the author(s) and the published article's title, journal citation, and DOI.

Supplement DOI: https://doi.org/10.6084/m9.figshare.30327376

Parent Article DOI: https://doi.org/10.1364/BOE.576585

Optical finger phantom with realistic optical properties: supplemental document

Experimental setup

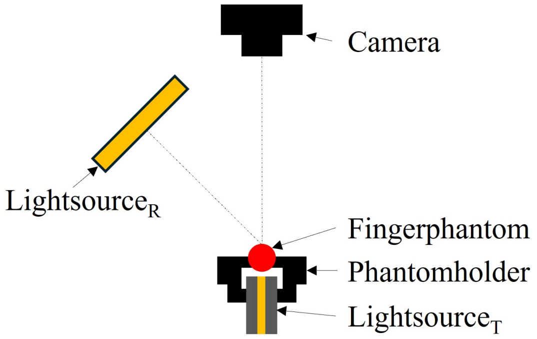

Figure S1: Schematic of the experimental setup used to compare images of a phantom and a human finger under above and beneath illumination. Lightsource$_R$ is a white LED panel (160×160 mm) positioned at 45° to the surface for above illumination. Lightsource$_T$ is a 5 mm diameter liquid light guide connected to a halogen lamp for beneath illumination. The phantom holder, fabricated from black SLA resin, accommodates both the phantom and Lightsource$_T$. The camera is positioned parallel to the surface.

Optical properties

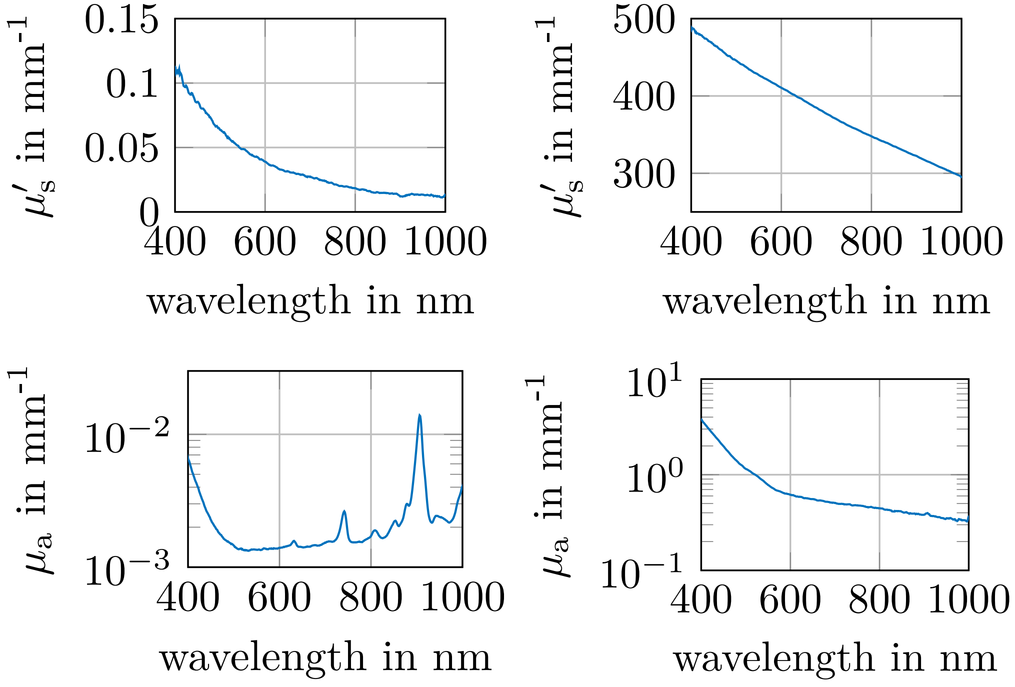

(a) (b)

Figure S2: Optical properties of the base material silicone (a) and the scattering particles zirconium oxide (b) [1].

References

[1] Markus Wagner, Oliver Fugger, Florian Foschum, and Alwin Kienle. Development of silicone-based phantoms for biomedical optics from 400 to 1550 nm. *Biomedical Optics Express*, 15(11):6561–6572, 2024.
